# Heart Rate Variability: A Measure of Cardiovascular Health and Possible Therapeutic Target in Dysautonomic Mental and Neurological Disorders

**DOI:** 10.1007/s10484-022-09572-0

**Published:** 2022-11-22

**Authors:** Martin Siepmann, Kerstin Weidner, Katja Petrowski, Timo Siepmann

**Affiliations:** 1 Department of Psychotherapy and Psychosomatic Medicine, University Hospital Carl Gustav Carus Dresden, Technische Universität Dresden, Fetscherstr. 74, 01307 Dresden, Germany; 2 Graefliche Kliniken, Bad Driburg, Germany; 3 Medical Psychology and Medical Sociology, University Medical Center of the Johannes Gutenberg - University Mainz, Mainz, Germany; 4 Department of Neurology, University Hospital Carl Gustav Carus, Technische Universität Dresden, Dresden, Germany

**Keywords:** Neurocardiac, Autonomic, Heart, Psychosomatic, Psychocardiology

## Abstract

Mental illness such as depression and anxiety as well as cerebrovascular disease are linked to impairment of neurocardiac function mediated by changes to the autonomic nervous system with increased sympathetic and decreased parasympathetic activity. Autonomic neurocardiac function can be evaluated by computing heart rate variability (HRV). Over the past decades, research has demonstrated the diagnostic value of HRV as independent predictor of cardiovascular mortality and as disease marker in progressive autonomic nervous system disorders such as Parkinson’s disease. Here we summarize our studies on HRV and its therapeutic modulation in the context of psychopharmacology as well as psychiatric and neurological disorders to honor the life of Professor Evgeny Vaschillo, the true pioneer of HRV research who sadly passed away on November 21st, 2020.

## Introduction

Our group has focused on heart rate variability (HRV) as a measure of cardiovascular and mental health across a wide range of research studies. This review article summarizes the body of evidence generated by our group highlighting its interdisciplinary implications in science and medical practice. This is to honor the life of Professor Evgeny Vaschillo, the true pioneer of HRV research who sadly passed away on November 21st, 2020.

### The Origins of HRV Research

Heart rate variability (HRV), beat to beat variation in the duration of the R-R interval—the heart rate period, is a marker of cardiac autonomic regulation. Historically, pulse rate was first measured by the ancient Greek physician Herophilus (335–280 BC) by timing with a water clock and introduced by Galen of Pergamon (131–200 AD) as a prognostic sign of various disease conditions (Billman, 2011). With the invention of galvanometers to record changes of electrical currents in the late nineteenth century and the development of advanced digital signal processing beginning in the 1960s, it became possible to evaluate cardiac rhythm on a beat- to beat basis (Shaffer et al., 2014). Power spectral analysis of heart rate was introduced in the early 1970s (Hyndman et al., 1971). Since then both time and frequency domain measures have been established to analyze HRV quantitatively (Task Force of the European Society of Cardiology & the North American Society of Pacing & Electrophysiology, 1996). According to Furlan and Barbic (2012) frequency domain analysis of heart rate and blood pressure variability has allowed detection of changes in the physiological basis of cardiovascular autonomic regulation that would have been hidden when considering the variations of heart rate and blood pressure by a time domain analysis. As an example, the frequency domain analysis of systolic arterial blood pressure reveals reduced values of low frequency systolic arterial blood pressure (LF_SAP_) compared to healthy subjects during a 75° head up tilt maneuver that is consistent with the presence of early and subtle abnormalities in the sympathetic vasomotor control in the patients who are free from orthostatic intolerance. Vaschillo et al. (2012) conceptualized the arterial baroreflex system (BRS) as a loop system with three components: heart rate, vascular tone and stroke volume counteracting acute shifts in blood pressure. A method allowing assessment of baroreflex function comprehensively has been introduced by his group as a tool to evaluate, diagnose and treat various cardiovascular diseases.

### The Link Between Poor Cardiovascular Health and Autonomic Dysfunction of the Heart in Chronic Mental Illness

The results of the womens’ health initiative study that followed more than 70,000 postmenopausal women without a history of cardiovascular disease over 4 years, demonstrate that depression symptoms at baseline significantly predict cardiovascular death after adjustment for age, race, education, income and cardiac risk factors (Wassertheil-Smoller et al., 2004). The INTERHEART study, a case control study of more than 11,000 first myocardial infarction cases and age and sex matched controls reported a population attributable risk (PAR) of about 9% associated with depression in the year previous to the myocardial infarction that is comparable to the PAR for myocardial infarction associated with diabetes (Frasure-Smith & Lesperance, 2006; Yusuf et al., 2004). It is well documented that depression predicts slow recovery and increases the mortality risk after acute myocardial infarction (Frasure-Smith et al., 1993). Kawachi et al. (1995) as well as Thayer et al. (1996) found increased mortality rates in patients with generalized and phobic anxiety and came to the conclusion that persons with chronic, severe anxiety may literally be worrying themselves to death. A possible explanation for impaired cardiovascular health and increased cardiovascular mortality in patients with chronic mental disorders might be a chronic disturbance of the functional integrity of the heart due to long-term dysregulation of the autonomic nervous system. In fact, common mental disorders such as depression and anxiety were found to be strongly associated with reduced HRV (Chalmers et al., 2016; Rechlin et al., 1994). Furthermore, state anxiety is associated with increased heart rate and reduced baroreflex control of heart rate in older adults with major depression (Watkins et al., 1999). In turn, a reduced HRV corresponds with low efficiency of autonomic control and an increased vulnerability to stress (Vaschillo et al., 2008). Diminished vagal control of the heart increases the vulnerability to sympathetically driven cardiovascular ischemia and malignant arrhythmias, contributing to elevated mortality. This pathophysiological interplay may be regarded as vicious circle where chronic mental illness impairs autonomic control of the heart and resulting impairment of cardiovascular health increases vulnerability to stress. It also highlights the potential importance and utility of HRV as diagnostic and therapeutic target to improve psychological and cardiovascular outcomes in patients with mental disorders.

### The Role of Heart Rate Variability and the Neuro-Hormonal System in Chronic Mental Illness

Excessive worrying is a common trans-diagnostic sign in mental illness that impairs cardiac autonomic balance through activation of the hypothalamic–pituitary–adrenal (HPA) system (Hu et al., 2019). It has been hypothesized that autonomic imbalance links depression and anxiety with an increased risk of cardiovascular morbidity and mortality risk (Musselman et al., 1998; Nemeroff et al., 1998). The immune system is activated with increased levels of proinflammatory cytokines in anxious and depressive mood states predisposing patients to develop artery stiffness and peripheral arterial hypertension (Sala et al., 2009). According to Carter and Tranel (2012) the process starts with an external stimulus i.e. a traumatic life event causing ruminative thinking that facilitate hypothalamic outputs to the heart via the vagus nerve with diminished parasympathetic influence over sympathetic control. A series of changes follows, including the release of noradrenaline from the locus coeruleus and corticotropin-releasing hormone from the hypothalamus (Kim et al., 2018) leading to elevated heart rate and blood pressure and decreased HRV. Although the mechanisms underlying the relationship between depression and cardiac events have not yet been fully elucidated neurohormonal dysregulation may partially explain the effects of depression on cardiovascular morbidity and mortality. Evidence includes elevated plasma and urinary catecholamines and cortisol found in medically well patients with major depressive disorder as well as electrophysiological alterations of cardiac function such as reduced threshold for ventricular arrhythmias (Carney et al., 2002).

### Therapeutic Modulation of HRV and Its Effect on Cardiovascular Outcomes in Patients with Chronic Mental Illness

Research conducted in the 1990s has suggested that modification of heart rate variability and baroreflex sensitivity by antidepressant treatment translates into cardiac protection (Vanoli et al., 1998) but large confirmatory trials are lacking to date. There is some evidence that treatment with cognitive behavioral or electroconvulsive therapy can improve impaired cardiac autonomic function and lower the risk of cardiovascular complications in depressed patients. It is however not clear if death rate however can be reduced by antidepressant treatment (Agelink et al., 2004; Nahshoni et al., 2004). It needs to be acknowledged that the effects of pharmacotherapy on cardiovascular autonomic function crucially depend on the class of substances used. In fact, antidepressants and antipsychotic compounds with tricyclic chemical structure (TCAs) may even increase the risk of cardiovascular morbidity and mortality. By contrast serotonin selective inhibitors (SSRIs) have been suggested to increase HRV and counteract the cardiovascular adverse effects of depression and anxiety (Cohen et al., 2000; Rechlin, 1995; Schmid et al., 2004). However, this effect has not translated into reduced cardiovascular mortality in patients with heart disease and depression in a large randomized controlled trial (Berkman et al., 2003). Vaschillo et al. (2008) developed a method to investigate the autonomic cardiovascular reaction to emotional picture cues and expectancy effects of alcohol consumption based on an 0.1 Hz HRV index. It was demonstrated by them (2002) that biofeedback produces oscillations in heart rate, blood pressure, vascular tone, and pulse amplitude via paced breathing at a specific resonance frequency. The group designed a HRV biofeedback method based on the baroreflex that may be useful as an adjunct treatment in various autonomic disorders and also in psychosomatic illness (Wheat & Larkin, 2010). While smaller studies showed overall promising results indicating that HRV biofeedback may improve psychiatric symptoms and increase HRV in patients with psychiatric and cardiovascular disorders, its effect on cardiovascular outcomes, especially mortality remains to be elucidated (Caldwell & Steffen, 2018; Chang et al., 2020; Climov et al., 2014; Hartogs et al., 2017; Lin et al., 2012; Penzlin et al., 2015, 2017, 2019; Siepmann et al., 2008a, 2008b, 2014). In summary, HRV might be altered by pharmacological and by non-pharmacological methods. Uncertainty remains with respect to the capacity of these means to decrease the risk of cardiovascular mortality in patients who have chronic cardiac dysautonomia due to mental illness.

In the following, our studies on the role of neurocardiac autonomic function in psychopharmacology as well as the effects of psychosocial stress on HRV in the context of cardiovascular health are presented. The results of HRV biofeedback studies in patients with depression, anxiety, addictive disorder and Parkinson’s disease are summarized.

## Review of Our Research and Findings

### HRV in Psychopharmacology

Antidepressant and anxiolytic agents have strong anticholinergic properties. Hence, these drugs can increase heart rate and prolong the QT interval through blockage of potassium ion channels of the heart leading to ventricular arrhythmias and sudden cardiac death (Du et al., 2019). Witchel et al. (2003) pointed out that differences in cardiovascular risk within classes of psychotropic drugs may result from pleiotropic cellular effects of the particular compounds that influence the drug-induced inhibition of repolarizing potassium current. Given the susceptibility of depressed patients to autonomic neurocardiac dysfunction clinicians should be aware of unwanted cardiac autonomic effects of antidepressant and anxiolytic medication. By contrast, pharmacological treatment of mental illness also may exert protective effects on the heart by ameliorating autonomic stress responses. Since the QT interval of the ECG depends on heart rate and individual duration of previous cardiac cycle length, researchers have focused on drug-induced changes in HRV that predict cardiovascular mortality (Sala et al., 2009). We have investigated the effects of subchronic dosing with various antidepressant drugs of different classes on HRV and other measures of autonomic functional integrity, including sudomotor and vasomotor assessment, as well as cognitive functions. These studies were conducted under randomized double-blind placebo-controlled cross-over conditions in healthy subjects. In addition, a paradigm to test the acute effects of anxiolytic drugs on autonomic responses to aversive stimuli was introduced. Pharmacological treatment may enhance mortality rates through unwanted cardiovascular effects. Treatment with psychotropic medication can cause sympathovagal imbalance and subsequently may increase the patients’ cardiovascular mortality risk (Celano et al., 2016; Maslej et al., 2017; Nemeroff et al., 1998; Vaccarino, 2000). The effects of pharmacotherapy on cardiovascular autonomic function deem to be class dependent. Antidepressants and antipsychotic compounds with tricyclic chemical structure (TCAs) increase the risk of cardiovascular morbidity and mortality most. By contrast serotonin selective inhibitors (SSRIs) have been suggested to counteract the detrimental cardiovascular adverse effects of depression and anxiety (Cohen et al., 2000; Rechlin, 1995; Schmid et al., 2004). However, treatment with SSRIs was not found to influence cardiovascular mortality in patients with heart disease and depression in a large randomized controlled trial (Berkman et al., 2003). Monoamine oxidase inhibitors (MAOIs), selective norepinephrine uptake inhibitor (SNRIs) frequently cause autonomic dysfunction. Herbal drugs such as hypericin are considered to be free any cardiovascular side effects. We aimed to assess the effects of psychotropic medication on cardiovascular autonomic regulation by measuring HRV in healthy humans.

#### Amitriptyline and St. John’s Wort Extract

Amitriptyline is a TCA that inhibits the reuptake of noradrenaline and serotonin from the synaptic cleft. The drug exerts autonomic and sedative effects due to its antimuscarinic, alpha-adrenolytic and antihistaminergic activity. Amitriptyline is therefore not recommended as a first-line drug to treat depression. We hypothesized that amitriptyline reduces HRV due to its pronounced anticholinergic activity.

St. John’s wort extract (*Hypericum perforatum*) is a phyto drug used by many cultures to treat depression. St John’s wort extract is widely promoted as a ‘natural antidepressant’ which lacks unwanted autonomic cardiac effects. It displays non-inferior efficacy in mild to moderate depression when compared to standard antidepressant drugs (Weixlbaumer et al., 2020). The pharmacological mechanism of the herbal drug has not been yet been understood despite known pleiotropic effects of the main psychoactive ingredients hypericin and hyperforin on the aminergic, GABAergic and glutamatergic neurotransmitter systems (Butterweck, 2003). St John’s wort extract inhibits the synaptic reuptake of noradrenaline. It exerts central cholinergic actions. We hypothesized that St. John’s wort increases HRV due to its cholinergic effects. Our observations indicated that amitriptyline decreases time and frequency domain measures of HRV and increases heart rate (HR). By contrast, St. John’s wort extract altered neither time domain measure of HRV nor HR. Amitriptyline decreased self-rated activity whereas St. John’s wort extract did not influence subjective mood (Siepmann et al., 2002). Neither amitriptyline nor St. John’s wort extract influenced cognitive performance. Amitriptyline but not St. John’s wort extract attenuated vasoconstrictory response of cutaneous blood flow (VCR) and skin conductance response (SCR) to sympathetic stimulation (Siepmann et al., 2004a, 2004b). In conclusion, patients receiving amitriptyline should be informed about possible drug induced cardiac side effects. The preexisting of cardiac conditions should be assessed also by ECG and a relevant prolongation of the QTc interval should be excluded prior to treatment (Schmid et al., 2004).

#### Sertraline

Sertraline is a selective serotonin reuptake inhibitor (SSRI). Like other substances of its class sertraline targets the serotonin system more selectively when compared to TCAs. However, dysautonomic symptoms i.e. symptomatic bradycardia and hypotension have also been noted with SSRIs (Siepmann et al., 2003). We hypothesized that sertraline increases HRV due to sympathoinhibitory properties. Multiple dosing with sertraline led to a reduction in heart rate whereas HRV, SCR, mood states and cognitive functions remained unchanged (Siepmann et al., 2003).

#### Moclobemide

Moclobemide exerts antidepressant activity through reversible and competitive inhibition of the A-form monoaminoxidase (MAO-A). Moclobemide has negligible anticholinergic and antihistaminergic activity. It should therefore not impair cognitive functions Autonomic side effects are noted less frequently when compared to irreversible inhibitors of MAO type A and B that decrease sympathetic activity to a high extent. Consistently, moclobemide did not change HRV nor SCR when given subchronically to healthy subjects in a previous study by our group (Siepmann et al., 2004a, 2004b). Cognitive performance remained unaltered whereas subjective tiredness was reduced in this investigation.

#### Venlafaxine

Venlafaxine is a selective serotonin and norepinephrine uptake inhibitor (SSNRI) with no significant affinity to cholinergic, histaminergic, adrenergic and dopaminergic receptors. We found sustained VR and shortening of the recovery time of the pupillary light reflex (Siepmann et al., 2007a, 2007b) consistent with norepinephrine reuptake blockade in cutaneous blood vessels and iris. By contrast, HRV and cognitive functions were noted unchanged (Siepmann et al., 2008a, 2008b). Measures of serum concentrations of venlafaxine and its main active metabolite, *O*-desmethylvenlafaxine, revealed rapid tolerance of the drug induced effect on the pupillary light reaction (Lindauer et al., 2008).

#### Reboxetine

Reboxetine is a selective norepinephrine (NE) reuptake inhibitor and shows only low affinity for adrenergic and muscarinic receptors (Agelink et al., 2002). Despite its pharmacological selectivity, autonomic disturbances such as dry mouth, constipation and difficult urination frequently occur during treatment with reboxetine (Siepmann et al., 2001). We therefore hypothesized that reboxetine impairs autonomic functions. We observed a prolonged dilation phase of VR and decreased SCR that may result from sympathetic activation and antimuscarinic activity of the antidepressant compound. By contrast, cognitive functions were not found altered (Siepmann et al., 2001).

#### Bupropion

Bupropion is an antidepressant of the aminoketone class (amphebutamone) that is considered to be relatively free of cardiac side effects (Siepmann et al., 2005a). Bupropion and its multiple active metabolites selectively inhibit reuptake of norepinephrine and dopamine (NDRI). The antidepressant activity of bupropion is achieved through its effects on the levels of norepinephrine and dopamine in the brain. We hypothesized that bupropion decreases HRV due its sympathomimetic activity. We observed that bupropion decreases HRV (root mean square of successive differences of RR-intervals) and increases heart rate when given for 14 days at daily doses of 300 mg to healthy subjects. One might therefore speculate that bupropion exerts anticholinergic action. Cognitive functions such as choice reaction, memory, psychomotor performance, flicker fusion frequency and subjective mood were not found influenced but drug induced reductions of absolute alpha and theta power density in the quantitative EEG (qEEG) hinting at a subtle psychostimulant effect were noted (Siepmann et al., 2005b).

#### Lorazepam

Fear can be targeted by benzodiazepines that enhance the inhibitory effect of the neurotransmitter γ-aminobutyric acid (GABA) at GABA (A) receptors. Lorazepam is a short acting benzodiazepine that dose-dependently acts anxiolytically and sedatively and impairs attention. In addition, the drug may influence autonomic regulation by central vagolysis (Siepmann et al., 2007a, 2007b). Our study aimed to assess the effects of lorazepam on autonomic responses to stressful visually and acoustically presented stimuli when given at non-sedative doses. Lorazepam significantly attenuated SCRs but not subjective feelings of anxiety to aversive versus neutral stimuli when given at single non-sedative doses.

### The Effects of Psychosocial Stress on Heart Rate Variability

Emotional reactions to psychosocial stress comprise changes in HR and HRV due to the activation of the sympathetic nervous system and inhibition of the parasympathetic nervous system. When stress becomes severe a hypothalamus–pituitary–adrenal (HPA) axis response ensues. The neuroendocrine reaction includes the release of corticotropin releasing factor (CRF), adrenocorticotropic hormone (ACTH), norepinephrine and cortisol secretion (Ziegler, 2012). Psychosocial stress can be defined as the individual’s response to a challenging environment such as unemployment (Andreassi, 2007). Factors determining how an individual copes with a stressful situation i.e. personality traits and mood states have to be considered when investigating neurocardiac responses to psychological stressors. The Trier Social Stress Test (TSST, Kirschbaum et al., 1993) was employed by our group in order to simulate real life stressors in a serious of studies. The standardized test includes a mock job interview with a free speech as well as an arithmetic task. The intensity of the experimental stress results from the personal relevance of the speech with high ego involvement, the extent of social-evaluative threat, and the anticipation of negative evaluation by a two-person panel (Dickerson et al., 2008). In order to investigate effects on the HPA axis reactivity a retest protocol has to be robust against possible habituation effects. We observed a significant habituation effect of the cortisol response to the TSST in healthy subjects undergoing retest after 24 h and complete restoration of HPA axis reactivity when retesting followed a 10 week test free week interval (Petrowski et al., 2012a, 2012b). We then went on and conducted a controlled trial in patients with panic disorder performing the TSST on repeated occasions. Patients showed a more pronounced impairment of HRV under testing conditions as compared to healthy subjects whereas baseline values did not differ between both groups (Petrowski et al., 2010). It can be suggested that increased sympathetic activation and/or parasympathetic inhibition during psychosocial stress conditions could be a marker of the disease. There are inconsistent findings on the reactivity of the HPA axis in patients with panic disorder. Normal levels of plasma cortisol, hypercortisolism as well as hypocortisolism have been reported (Abelson et al., 2007; Garcia-Leal et al., 2005; Petrowski et al., 2010). While the major HPA and immune system effect of depression and anxiety has not been fully elucidated yet, it seems to be dominated by inflammation with liberation of inflammatory interleukin correlates to prepare the body for invasion. In depression active coping decreases, so the body prepares for the worst. The corticoid effect seems to be an anti-inflammatory homeostatic response, activated by the same processes that produces inflammation. Sometimes the inflammatory process prevails, suppressing anti-inflammatory effects, and sometimes the anti-inflammatory effect dominates. In the latter case, corticoid effects may dominate.

A study of our group found complete unresponsiveness to psychosocial stress in patients with acute and remitted panic disorder (Petrowski et al., 2011, 2012). Introduction of interoceptive stress by means of the dexamethasone-corticotropin-releasing-hormone (DEX-CRH) test into the TSST paradigm provoked a less pronounced decrease in HRV in patients with panic disorder as compared with healthy controls (Petrowski et al., 2017). Hyporeactivity of the HPA axis is a known marker of depression. It is hypothesized to reflect pathophysiologic changes at the central nervous system (CNS) level (The APA Task Force on Laboratory Tests in Psychiatry, 1987). Therefore, the blunted response of the neurocardiac autonomic system to the dexamethasone-corticotropin-releasing-hormone (DEX-CRH) test could be due to a high proportion of comorbidly depressed patients (64%) in our patient sample. It has been demonstrated that occupational stress may enhance the risk of cardiovascular morbidity and mortality through neurocardiac autonomic dysregulation (Sara et al., 2018). The stress load of emergency service employees is particularly high due to continuous exposure to traumatic incidents and shift work. However, individual physiological reactions to critical incidents are heterogenous and assessment tools that enumerate encounters resulting in distress are needed (Boland et al., 2018). We investigated the physiological stress level of emergency physicians of the helicopter emergency service between emergency operations on one shift. We found decreased HRV, increased heart rate and elevated self-perceived stress level on the air rescue days. These changes were most pronounced between the alarm and landing phase of the emergency operations. Complete recovery was noted in between the air rescue days that is interpreted by us a sign of regeneration ability (Schöniger et al., 2020a, 2020b).

Distinctive personality characteristics may be linked to mood states that reduce HRV and negatively influence cardiovascular morbidity i.e. a type A behavior pattern that is characterized by impatience, competitiveness, and hostility increases an individual’s risk to develop coronary artery disease (Friedman, 1989). It has been proposed that one mechanism contributing to coronary artery disease is excessive responsivity of the neurocardiac autonomous nervous system to environmental stressors. Type D personality is characterized by negative affectivity and social inhibition. Persons with type D personality are less overtly aggressive than those with type A. They tend to experience more toward introspection and are inclined to perceive selectively the negative sides of themselves and their environment (Andreassi, 2007). It is imaginable that this personality pattern can be found more frequently among the unemployed since it is difficult to place introverted, socially inhibited and persons who are anxious and dysphoric on the job market (Petrowski et al., 2017). It could reduce HRV and trigger coronary artery disease. We found 53% type-D personality pattern in a community sample of unemployed subjects. In reference, prevalence of type-D-personality in the normal population is indicated between 9 and 33% for European countries % (Grande et al., 2004). Type-D-personality was noted by us to be significantly associated with depressiveness, low self-esteem and physical complaints. By contrast, HRV did not differ between individuals with and without type-D-personality when undergoing stress tests. In line with the results of our study, Kang et al. (2015) could not find an association between HRV and type D personality pattern in participants of a community-based mental health program.

### Heart Rate Variability as Disease Marker in Neurodegenerative α-Synucleinopathies

Dysautonomia is seen in almost all neurodegenerative disorders compromising quality of life of affected patients in various ways. The α-synucleinopathies are neurodegenerative disorders that display accumulation of misfolded α-synuclein aggregates in glial cells as well as in neurons (Mendoza-Velásquez et al., 2019). The group of α-synucleinopathies include Parkinson’s disease, dementia with Lewy bodies, multiple system atrophy and multiple rare neuroaxonal dystrophies. Pure autonomic failure (PAF) is a nowadays considered by many authors neurodegenerative α-synucleinopathy too as it features severe generalized dysautonomia as well as cytoplasmic α-synuclein inclusions in postganglionic small autonomic small nerve fibers and is linked to a high-risk condition of developing Parkinson’s disease, dementia with Lewy bodies, or multiple system atrophy in later life (Kaufmann et al., 2017). The autonomic nervous system has been identified a promising diagnostic and therapeutic target in patients with α-synucleinopathies as they frequently display dysautonomic symptoms that often even precede motor dysfunction (Palma, 2018). Dysautonomia in these patients can manifest with reduced heart rate variability and various symptoms including orthostatic hypotension, constipation, urinary and sexual dysfunction amongst others. Studies of neurocardiac function shed light on the possible utility of HRV analysis in different α-synucleinopathies. A study by our group showed that in patients with early Parkinson’s disease, HRV is not different compared to healthy control subjects whereas pilomotor, sudomotor and vasomotor autonomic skin function shows substantial impairment (Siepmann et al., 2016). This observation is consistent with previous research that revealed stable HRV in early Parkinson’s disease and deterioration in later stages of the disease (Maetzler et al., 2009). However, it needs to be acknowledged that the literature on neurocardiac function in Parkinson’s disease includes partially contradictory reports. For instance, results from an observational cohort study demonstrated elevated risk of Parkinson’s disease in people with low HRV, which in turn might suggest that HRV is reduced in prodromal disease stages (Alonso et al., 2015). More than half of dementia with Lewy bodies and multiple system atrophy patients can develop dysautonomic symptoms before symptoms of compromised motor and cognitive function occur (Bhatia & Stamelou, 2017; Coon et al., 2018). Cardiovascular dysautonomic symptoms like orthostatic hypotension and attenuated heart rate variability are frequent in these patients and lead to a substantial reduction in quality of life (Norcliffe-Kaufmann et al., 2018). However, the body of evidence on the utility of HRV analysis in α-synucleinopathies is still scarce substantiating a need for large prospective observational studies.

### Heart Rate Variability in the Context of Hypertensive Pregnancy Disorders and Preterm Birth

While the predictive value of HRV in the context of cardiovascular risk is well described, uncertainty remained on its applicability to pregnancy disorders that have long-term offspring cardiovascular sequelae (Siepmann et al., 2014). Interestingly, reduced HRV was observed in infants born preterm with attenuation being greater in those who display higher scores on clinical illness scales (Goldstein et al., 1998; Patural et al., 2008). Pregnancy complicated by preterm delivery or a hypertensive pregnancy disorder such as preeclampsia are linked to an elevated risk of cardiovascular events in later life (Lewandowski et al., 2015). The offspring of these women spring show a distinct cardiovascular phenotype, which is hallmarked by rarefaction of small vessels and hypertrophy of the heart (Lewandowski et al., 2013; Yu et al., 2016). These structural patterns of cardiovascular system changes emerge during the first 3 months of life, coinciding with the development of changes in HRV. A study with our group contributed to therefore assessed HRV in 98 sleeping neonates from the EPOCH program (Effect of Pregnancy on Offspring Cardiovascular Health study). At the time of birth assessment babies underwent a short 5–10-min electrocardiogram while they were lying down between feeds without use of a pacifier. This study demonstrated that increasing prematurity, but not hypertension of the mothers’ during pregnancy was linked to attenuated HRV at birth (Aye et al., 2018). Moreover, babies born preterm displayed sympathovagal imbalance when compared to term infants. However, there was no association between differences in cardiac autonomic function at birth and measures of cardiovascular structural and functional integrity such as vessel density, ejection fraction or pulse wave velocity 3 months post birth.

Some uncertainty remained regarding the consequences of dysautonomia in hypertensive pregnancy and preterm birth. Another study with contribution from our group was able to demonstrate that young women with a history of preeclampsia display changes to cerebral structure including temporal lobe white matter changes (Siepmann et al., 2017) The severity of changes to cerebral structure was found proportional to time since pregnancy indicating continued progress of damage after the pregnancy. Moreover, these changes could not be explained by the mothers’ classic cardiovascular risk profiles suggesting that preeclampsia might pose an independent risk factor of structural brain change in later life. The pathophysiological mechanisms of brain changes in previously preeclamptic women might include placental dysfunction, with widespread endothelial dysregulation and attenuated brain perfusion or dysautonomia leading to impaired cerebrovascular hemodynamics.

Taken together, a link between impaired autonomic function and hypertensive pregnancy disorders seems probable but the exact underlying pathophysiology as well as the predictive and diagnostic value of HRV in this population remains to be elucidated. This research gap was further substantiated by a systematic review by our group, which identified 26 studies providing data from 1854 pregnant women (Yousif et al., 2019) In this synthesized population of young women, 453 had preeclampsia and 93.6% of these (n = 424) showed objectifiable signs of autonomic dysfunction. Eleven out of the 26 studies included reported on HRV. While three of these investigations found no differences on HRV analysis between preeclampsia and normotensive pregnancy, eight studies reported HRV changes consistent with an increase in sympathetic activity and suppressed parasympathetic tone in preeclamptic women. This heterogeneity among observations highlights the need for large prospective cohort studies to define the role of HRV in hypertensive pregnancy.

### HRV Biofeedback: Non-invasive Autonomic Neuromodulation

**Fig. 1:**
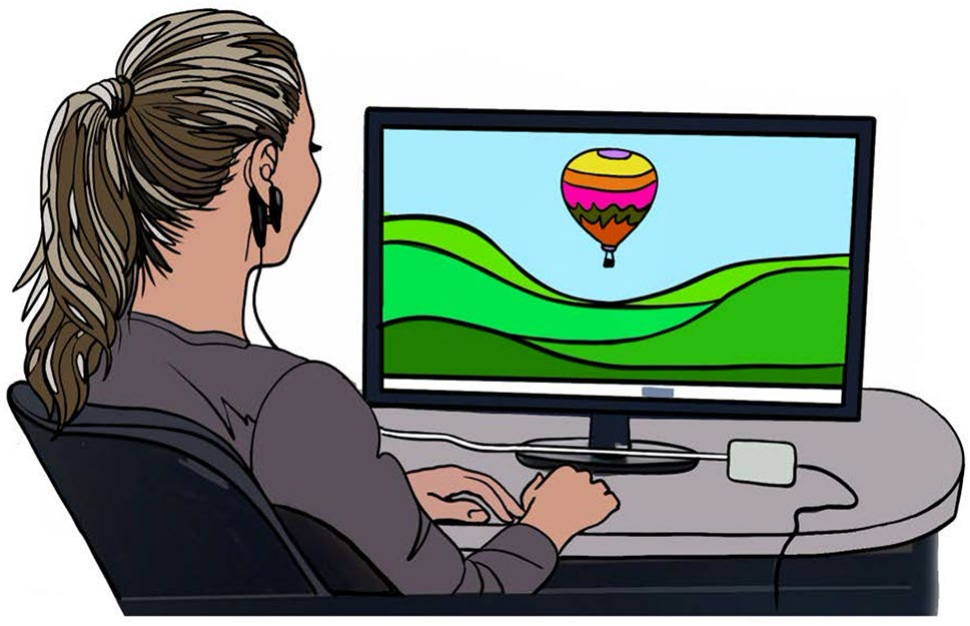
Heart rate variability biofeedback training. Illustration of a subject undergoing HRV biofeedback. The heart rate is continuously captured using an ear clip pulse oximetry sensor and HRV is computed in real time with visualization on the screen (balloon). Image by Antje Siepmann

HRV biofeedback is a non-invasive, non-pharmacological treatment technique, which employs a metronomic breathing technique to increase parasympathetic activity and in consequence HRV. During biofeedback training HRV is visualized as moving object in real time on a computer screen to provide visual feedback on treatment success. A common visualization is a balloon or butterfly that ascends with increasing HRV and descends with decreasing HRV as illustrated in Fig. 1.

HRV biofeedback enhances the signal input intensity by vagal afferent nerve stimulation conveyed via the nucleus tractus solitary (NTS) and projected to cortical, paralimbic and limbic structures of the brain, known to be involved in emotional regulation (Pinter et al., 2019). It has been suggested that HRV biofeedback may improve interoceptive representation of the insular cortex (Hodossy & Tsakiris, 2020), therefore reduce alexithymia and ameliorate somatic symptoms and difficulties in concentrating and decision-making in common mental disorders such as depression. The HRV biofeedback technique requires cognitive focusing on nuances in breathing. Similar to mindfulness meditation ruminating can be interrupted (Lehrer & Gevirtz, 2014). The acquired skill to reduce heart rate in emotionally challenging situations by means of vagal control and the inhibition of sympathetic arousal exerts an anxiolytic effect and improves self-efficacy (Nolan et al., 2005).

### HRV Biofeedback: Signals of Efficacy in Dysautonomic Psychiatric and Neurological Disorders

#### Major Depressive Disorder

An open-label study by our group included 14 patients with major depressive disorder (MDD) aged 30 years (18–47) and 24 healthy subjects (Siepmann et al., 2008a, 2008b). Patients already receiving antidepressant and/or psychotherapy were enrolled. Half of the control subjects participated in three HRV biofeedback sessions per week over 2 weeks, half of them underwent an active control condition. Depression as assessed by the Beck Depression Inventory (BDI) was decreased in patients with depression at the end of the intervention and at 2 weeks follow-up. Reduced heart rate and increased HRV was noted at follow up. By contrast, there was no change in healthy controls receiving HRV biofeedback. To the best of our knowledge, there are three more published studies on the effects of HRV biofeedback in patients with MDD (Caldwell & Steffen, 2018; Hartogs et al., 2017; Karavidas et al., 2007). Karavidas et al. noted improvement of depressive symptoms and increases in HRV in 11 middle-aged patients. Hartogs et al. observed in seven patients increases in HRV following the biofeedback intervention whereas depressive symptoms remained unaltered. Both studies lacked a control group. Caldwell et al. performed a randomized-controlled study in 20 college students with MDD receiving conventional psychotherapy. They stated improvement of depressive symptoms as well as increases in HRV in participants receiving HRV biofeedback as an adjunct treatment as compared to those who received psychotherapy alone. While the aforementioned studies reported beneficial effects of HRV biofeedback on neurocardiac function in patients with MDD, large confirmatory trials are lacking to date as pointed out in a review (Pinter et al., 2019).

#### Panic Disorder

The pathophysiology of panic disorder is characterized by HRV reduction, hyperarousal and impaired adaptation to repeated stimuli (Zhang et al., 2020). The patients’ risk of cardiovascular morbidity and sudden cardiac death is increased (Härter et al., 2003; Shibeshi et al., 2007). HRV biofeedback targets sympathovagal imbalance and can alleviate hyperarousal (Lehrer et al., 2022). Results from a meta-analysis gives hint that HRV biofeedback has favorable effects in patients with panic disorder (Chalmers et al., 2014). A previous randomized controlled study by our group assessed the effects of HRV biofeedback in 52 two middle aged patients with panic disorder. Patients receiving a 4 week HRV biofeedback protocol with 12 sessions showed an increase in HRV and reduced anxiety whereas those undergoing an active control condition remained unchanged (Herhaus et al., 2022).

#### Alcohol Addiction

Patients with alcohol use disorder show HRV reduction and hypervigilance (Campanella et al., 2009; Quintana et al., 2013). Sympathoexcitatory responses to goal directed and environmental stimuli are insufficiently inhibited since the integrity of central autonomic network (CAN) of the patients’ brain regions is compromised (Chalmers et al., 2014). Stress as environmental or internal cues may thus lead to craving and vulnerability to relapse (Teeravisutkul et al., 2019).

We conducted a randomized controlled trial in 48 patients with alcohol dependence aged between 25 and 59 years undergoing an inpatient rehabilitation program in order to test the hypothesis that HRV biofeedback decreases craving and might be useful to supplement other treatment regiments (Penzlin et al., 2015). In the treatment group, patients attended 6 sessions of HRV biofeedback over 2 weeks in addition to standard rehabilitative care, whereas, in the control group, subjects received standard care only. Psychometric testing for craving (Obsessive Compulsive Drinking Scale), anxiety (Symptom Checklist-90-Revised), HRV assessment using coefficient of variation of R–R intervals (CVNN) analysis, and vasomotor function assessment using laser Doppler flowmetry were performed at baseline, immediately after completion of treatment or control period, and 3 and 6 weeks afterwards. Psychometric testing showed decreased craving in the biofeedback group immediately postintervention, whereas craving was unchanged at this time point in the control group. Anxiety was reduced at follow-ups three and six post-biofeedback but was unchanged in the control group. Following biofeedback, CVNN tended to be increased, albeit the change did not reach statistical significance. There was no such trend in the control group. Vasomotor function assessed using the mean duration to 50% vasoconstriction of cutaneous vessels after deep inspiration was improved following biofeedback immediately post-intervention and remained unchanged in the control group. A follow up survey conducted 1 year after completion of the trial gave hint for a possible increase in long-term-abstinence after HRV biofeedback as an adjunct to the rehabilitation program (Penzlin et al., 2017).

#### Preterm Labor

Preterm birth is a frequent complication of pregnancy that is linked to increased mental stress and dysregulation of the autonomic nervous system (Rich-Edwards & Grizzard, 2005). A randomized controlled study by our group aimed at counteracting both stress and dysautonomia in women with preterm labor. An improvement in neurocardiac function with elevated HRV was observed in women who underwent the biofeedback intervention but not in those who had been allocated to the control group. However, this beneficial effect on the autonomic regulation of the heart has not translated into the rate of preterm birth (Siepmann et al., 2014).

#### Acute Ischemic Stroke

Neurocardiac dysregulation with low HRV is linked to poor clinical outcome and increased cardiovascular mortality in patients who survived an acute ischemic stroke (Mäkikallio et al., 2004). This is relevant on a large scale as stroke is the second leading cause of death and a major cause of lasting disability in adults worldwide and more than three quarters of stroke survivors display symptoms due to compromised regulation of the cardiovascular system and other organs by the autonomic nervous system (Katan & Luft, 2018; Xiong et al., 2012, 2014). Our group recently performed a randomized sham-controlled study of HRV biofeedback in 48 stroke survivors who underwent standardized stroke unit care. In this trial we were able to demonstrate that integrating HRV biofeedback can be integrated in multidisciplinary standard stroke unit care and leads to improvement in neurocardiac function post stroke as well as and sustained alleviation of dysautonomic symptoms 3 months after the intervention (Siepmann et al., 2021). This observation was in line with another report of HRV biofeedback after stroke. This randomized study included stroke patients within a week from stroke onset. The results of this study were consistent with improved neurocardiac function following HRV biofeedback further lending support to the potential value of this treatment in stroke care (Chang et al., 2020).

#### Cardiovascular Disease

A systematic review identified 12 studies that reported on HRV biofeedback in patients with known cardiovascular disease (comprising arterial hypertension, heart failure or coronary artery disease) out of which nine had a randomized controlled design (Burlacu et al., 2021) Synthesized analysis of these studies suggested possible beneficial effects on readmission rates, blood pressure and left ventricular ejection fraction but most of the included studies were limited by overall small sample sizes (Bernardi et al., 2002; Chen et al., 2016; Climov et al., 2014; de Albuquerque Cacique New York, et al., 2021; Jones et al., 2010, 2015; Joseph et al., 2005; Lin et al., 2012; Nolan et al., 2010; Swanson et al., 2009; Yu et al., 2018) However it needs to be highlighted that some of these studies had substantially larger sample sizes than those reported in mental and neurological disorders and most of them reported benefits on objective clinical outcomes that exceed a positive effect on neurocardiac function as indicated by elevated HRV alone.

## Discussion

### The Diagnostic and Prognostic Value of HRV in the Context of Cardiovascular and Mental Illness

The potential value of HRV analysis in estimating cardiovascular risk has been pointed out by the task force of the European Society of Cardiology and the North American Society of Pacing and Electrophysiology more than a quarter century ago (Task Force of the European Society of Cardiology & the North American Society of Pacing & Electrophysiology, 1996). Ever since various studies have been undertaken to elucidate the diagnostic and predictive value of HRV and its time domain and frequency domain components. A substantial body of evidence was accumulated indicating that the measure can predict cardiovascular morbidity and even death in internal, neuropsychiatric and mental disorders. It is hypothesized by Bassi and Bozzali (2015) that an impaired baroreflex function predicts cognitive decline in patients with Parkinson disease, dementia, and related disorders and even in aging people that do not suffer from a neurodegenerative disease. In psychopharmacology heart rate analysis can be employed as a tool to detect drug induced autonomic neurocardiac dysregulation. Treatment with psychotropic agents exerting relevant autonomic toxicity such as atropine like tricyclic antidepressant agents can be monitored in patients with an enhanced risk for autonomic neurocardiac dysfunction. It is however not clear whether SSRIs and antidepressant compounds of other classes may positively influence or deteriorate autonomic neurocardiac dysregulation. Further research is needed to provide a risk–benefit analysis with threshold of risk for each drug through pharmacokinetic/pharmacodynamic studies adequately scaled and properly conducted to establish a therapeutic window as suggested by Sala et al. (2009).

### Research Gaps and the Journey of HRV Research: There is Still a Long Way to Go

A link between negative emotions such as anxiety and reduced HRV has been stated. HRV is found diminished in patients diagnosed with depression but not in Parkinson’s disease patients. Impairment of HRV by experimental induction of psychosocial stress is described in patients with Parkinson’s disease and in depressed individuals. Long term studies are needed to assess the effects of enduring work-related stress on HRV. Future studies should address the influence of comorbid depression on HRV in patients suffering from Parkinson’s disease. The influence of personality traits such as a type D pattern on neurocardiac autonomic regulation should be considered by means of long-term HRV measurements.

Findings on the activation pattern of the HPA axis connecting the heart and the brain in response to psychosocial stress conditions are less consistent in patients with such common mental disorders. The results of our studies suggest that a hyporesponsiveness of the HPA axis to psychosocial stressors indicates an enhanced risk for relapses in panic disorder after successful psychotherapy. Future studies should consider the pretreatment HPA axis reactivity under standardized psychosocial stress in order to constitute the HPA axis reactivity as possible vulnerability factor for the prediction of symptom remission and relapse following treatment (Petrowski et al., 2012a, 2012b).

Observational studies on measures of HRV in determining ANS function in patients with neurodegenerative α-synucleopathies have produced inconsistent findings. Since cardiovascular dysautonomic symptoms frequently occur and may negatively impact quality large scale prospective trials are needed. There is also a need for large prospective cohort studies to define the role of HRV in hypertensive pregnancy disorders such as preeclampsia.

## Conclusion

Taken together, the clinical use of HRV analysis in everyday practice is still limited to specialized centers with the exception of a few specific conditions that are associated with a high risk of malignant cardiac arrhythmia such as the Guillain–Barré syndrome (Meena et al., 2011). Why is that so? Possible explanations are manifold, ranging from inter-individual variability, susceptibility toward environmental factors to the multitude of individual health-conditions that may alter HRV. Moreover, one might wonder how, despite a strong independent association with mortality, little attention has been given to modifying HRV directly. Adding HRV biofeedback to psychotherapy can increase heart rate variability and augment treatment effects in various mental disorders and neuropsychiatric diseases (Caldwell & Steffen, 2018). We showed that HRV biofeedback can reduce chronic stress in patients with preterm labor when administered as an adjunct to routine care. While HRV effects are immediate, therapeutic effects are delayed. Several smaller scope studies demonstrated the beneficial effects of HRV biofeedback on HRV and autonomic function in prevalent disorders such as depression and stroke but confirmatory large randomized controlled trials are still lacking.

Analysis of HRV remains a promising target to characterize cardiovascular health in individual patients and modulate neurocardiac function but translating this potential into an objective improvement of cardiovascular outcomes will require additional large-scale confirmatory randomized controlled trials as well as real world data analyses. This effort would be necessary to understand whether modulation of HRV via biofeedback or other interventions is strong enough to alter the course of mental illness and improve cardiovascular outcomes in these patients as well as in patients who suffer from primary cardiovascular disorders. In our view, this will be one of the most important questions in HRV research in the future.
